# Which Prosthesis for Aortic Valve Replacement?∗

**DOI:** 10.1016/j.jacadv.2023.100402

**Published:** 2023-06-30

**Authors:** Hartzell V. Schaff

**Affiliations:** Department of Cardiovascular Surgery, Mayo Clinic, Rochester, Minnesota, USA

**Keywords:** AVR, bioprosthesis, mechanical valve, survival

**Figure:**
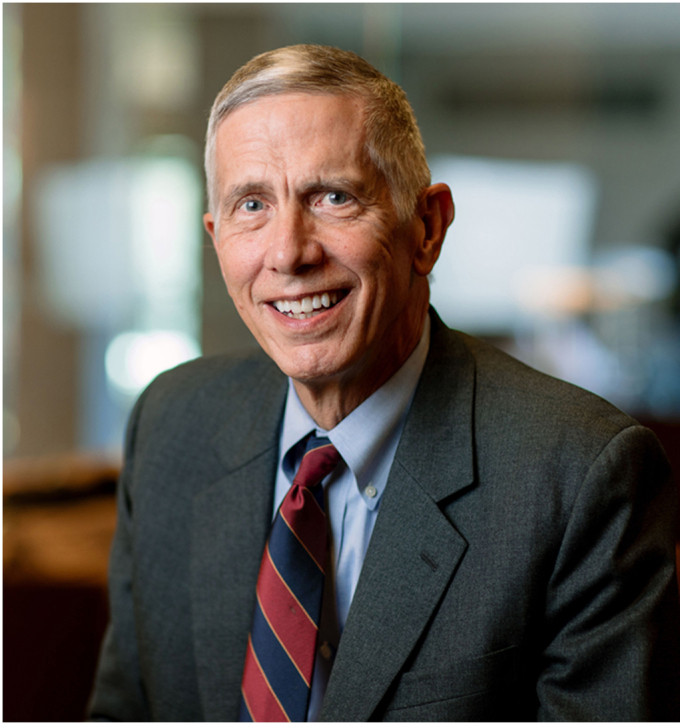

In 2016, Dr Glaser and colleagues published a report of patients aged 50 to 69 years who had undergone aortic valve replacement (AVR) in Sweden from 1997 to 2013.^1^ The study analyzed data from a national registry (SWEDEHEART [Swedish Web-System for Enhancement and Development of Evidence-Based Care in Heart Disease Evaluated According to Recommended Therapies]) of more than 4,000 patients who received mechanical valves (60%) or bioprostheses. In propensity score-matched patients, 10- and 15-year survivorship was higher in patients with mechanical valves compared to those with a bioprosthesis, 79% and 59% versus 75% and 50% (hazard ratio: 1.34; *P* = 0.006). Not unexpectedly, the risk of major bleeding was greater in patients with mechanical prostheses receiving warfarin, but the late risk of stroke was similar comparing the 2 valve categories. These results were generally similar to other studies of outcomes of AVR with mechanical and biological valves from the United States,^2^, ^3^, ^4^, ^5^ Canada,^6^ Switzerland,^7^ and the United Kingdom.^8^

Defining the long-term safety and efficacy of aortic valve substitutes will necessarily involve consideration of patient age and predicted longevity. It is generally accepted that durability of bioprostheses is adequate for older patients with limited life expectancy. But therein lies the rub, what is older, and what is limited life expectancy? Although the survival of patients after AVR is reduced compared to a “normal” population, the projected longevity for a 50-year-old male in the United States is 28 years, and for a female, it is 32 years^9^; the average life expectancy of a 70-year-old is 15 years.^10^ So, have improvements in bioprosthetic valve design and manufacturing and the availability of valve-in-valve transcatheter AVR changed the calculus in the selection of a prosthesis?

The most recent generation of stented heterograft valves for surgical AVR are constructed of bovine tissue, and several models are available commercially. In another study from the SWEDEHEART registry, Persson et al evaluated late survival and incidence of reoperation among patients undergoing AVR with various bioprostheses in a contemporary era. The Perimount valve was associated with the lowest incidence of reintervention, all-cause mortality, and hospitalization for heart failure.^11^ The question then follows whether the survival advantage of AVR with a mechanical valve in patients <70 years of age would be mitigated compared to the widely used Perimount bioprosthesis.

In this issue of *JACC: Advances*, Lu et al^12^ explored the outcomes of surgical AVR with the Perimount bioprosthesis compared to bileaflet mechanical valves, again using data from the SWEDEHEART registry and the Swedish National Patient Register. They analyzed 6,907 patients age 50 to 69 years (Perimount group, n = 3,831) and also performed subgroup analyses of patients 50 to 59 years and 60 to 69 years of age. At 15 years of follow-up, the estimated cumulative all-cause mortality was approximately 8% greater in the Perimount group than in the mechanical valve group (45%, 95% CI: 42%-48% vs 37%, 95% CI: 35%-40%). The survival advantage at 15 years with mechanical valves was less in patients 60 to 69 years of age, but among those age 50 to 59 years, survival with a mechanical valve was 15% greater 15 years postoperatively compared to Perimount valves. Late stroke and heart failure risks were similar among the prosthetic valve groups. The risk of late reintervention was lower, but the cumulative risk of bleeding was greater with mechanical valves.

In the discussion, the authors present a balanced acknowledgment that some studies comparing outcomes of AVR with mechanical valves and bioprostheses have found no difference in late survival in the 59- to 69-year age group. But it is worth noting that if there is true equipoise in outcomes with the 2 categories of prostheses, one might expect, in addition to studies such as this demonstrating survival benefit with mechanical valves, other large studies demonstrating superior outcomes with bioprostheses. There are very few such reports.

So what are the possible explanations for better overall survival with mechanical valves used for AVR? In retrospective database studies, there is the possibility of selection bias due to unrecorded clinical and hemodynamic variables, and it is possible that mechanical valves are utilized preferentially in patients with longer life expectancy and that bioprostheses may be selected in any age group when survival is thought to be limited. This may be true to a certain extent, but we more frequently encounter healthy young patients who insist on a bioprosthesis due to the perception that by avoiding anticoagulation with warfarin, they would be less limited in physical activity and lifestyle.

The intrinsic performance of the prosthesis or some benefit of chronic anticoagulation may contribute to a survival difference between the valve types. However, the hemodynamic performance of currently available bileaflet mechanical valves and bioprosthetic valves (porcine and pericardial) is generally similar, and it seems unlikely that any small difference in the function of normal prostheses would translate into an important survival benefit.

Many will point to the hazard of late reoperation in patients with a bioprosthesis and suggest that this will be mitigated by transcatheter valve insertion for degenerating valves. However, the mortality associated with reoperation on bioprostheses is relatively low,^13^ and deaths due to reoperation cannot account for the difference in late survival. Indeed, Weber et al^7^ reported that reoperation rates were not significantly different in their propensity-matched study of mechanical and bioprostheses for AVR, but late survival was superior among patients with mechanical valves.

A more likely explanation for better survival of patients age 50 to 69 years with mechanical valves is the hemodynamic consequences of living with a failing bioprosthesis. Although primary tissue failure of bioprostheses may progress rapidly (eg, cusp tear), many patients will have months or years of exposure to hemodynamically significant valvular regurgitation, stenosis, or both before critical prosthetic failure is identified, and replacement is advised. The impact of valve failure on mortality is underestimated by rates and risks of reoperation.^14^

Current American College of Cardiology/American Heart Association practice guidelines emphasize the importance of shared decision-making in selecting a prosthesis for AVR in patients age 50 to 65 years, emphasizing the trade-offs between durability (and need for reintervention), bleeding, and thromboembolism. But as demonstrated again in the present article, there appears to be survival benefit associated with the use of a mechanical prosthesis for AVR in this age range. Clinicians should discuss this potential advantage of mechanical valve substitutes with patients to inform their decision on valve selection fully.

The conundrum regarding prosthesis selection for AVR will likely continue as physicians and surgeons have certain biases about patient age and their expectations post-AVR. But the question of which valve is best for a given age group can be stated differently. Is there a patient age group for which a mechanical valve is the wrong option? Survival of young patients with mechanical AVR has resulted in many patients ≥70 years of age who live relatively normal lives with the prosthesis and warfarin anticoagulation,^15^ and studies show no difference in quality of life comparing elderly patients who have had mechanical valves or bioprostheses.^16^^,^^17^

## Funding support and author disclosures

The author has reported that he has no relationships relevant to the contents of this paper to disclose.
